# A *Pinus strobus* transcription factor *PsbHLH1* activates the production of pinosylvin stilbenoids in transgenic *Pinus koraiensis* calli and tobacco leaves

**DOI:** 10.3389/fpls.2024.1342626

**Published:** 2024-01-18

**Authors:** Yi Rae Kim, Jung Yeon Han, Yong Eui Choi

**Affiliations:** Department of Forest Resources, College of Forest and Environmental Sciences, Kangwon National University, Chuncheon, Republic of Korea

**Keywords:** transcription factor, bHLH, stilbene, methylated pinosylvin, overexpression of PsbHLH1

## Abstract

Transcription factors (TFs) play an important role in regulating the biosynthesis of secondary metabolites. In *Pinus strobus*, the level of methylated derivatives of pinosylvin is significantly increased upon pine wood nematode (PWN) infection, and these compounds are highly toxic to PWNs. In a previous study, we found that the expression of a basic helix-loop-helix TF gene, *PsbHLH1*, strongly increased in *P. strobus* plants after infection with PWNs. In this study, we elucidated the regulatory role of the *PsbHLH1* gene in the production of methylated derivatives of pinosylvin such as pinosylvin monomethyl ether (PME) and dihydropinoylvin monomethyl ether (DPME). When *PsbHLH1* was overexpressed in *Pinus koraiensis* calli, the production of PME and DPME was significantly increased. Overexpression of the stilbene synthase (*PsSTS*) and pinosylvin methyl transferase (*PsPMT*) genes, known as key enzymes for the biosynthesis of methylated pinosylvins, did not change PME or DPME production. Moreover, PME and DPME were not produced in tobacco leaves when the *PsSTS* and *PsPMT* genes were transiently coexpressed. However, the transient expression of three genes, *PsSTS*, *PsPMT*, and *PsbHLH1*, resulted in the production of PME and DPME in tobacco leaves. These results prove that *PsbHLH1* is an important TF for the pinosylvin stilbene biosynthesis in pine plants and plays a regulatory role in the engineered production of PME and DPME in tobacco plants.

## Introduction

Plants produce polyphenolic compounds called stilbenes, which are called phytoalexins and are known to have strong antibacterial and antioxidant activity (Hart, 1981). Pinosylvin-type stilbenes are produced mainly by *Pinus* spp. The content of pinosylvin and its methylated derivatives is absent or very low in young branches and leaves of pine plants, and it has been reported that the synthesis of these substances increases in response to biotic and abiotic stimuli (Gehlert et al., 1990; Harju et al., 2009; Valletta et al., 2021). On the other hand, methylated derivatives of pinosylvin are present in high concentrations in heartwood, a nonliving tissue, and are known to prevent wood decay, mainly by fungi (Harju and Venäläinen, 2006). Resveratrol stilbene, a compound produced by dicotyledonous plants such as grapes and peanuts, has been reported to exhibit a wide range of biological activities (Burns et al., 2002; Baur and Sinclair, 2006; Zhou et al., 2021; Bakrim et al., 2022). Due to the high industrial value of stilbene compounds, protocols have been developed to produce resveratrol and pinosylvin in bacteria and yeast using metabolic engineering systems (Halls and Yu, 2008; Jeandet et al., 2010; van Summeren-Wesenhagen and Marienhagen, 2015; Thapa et al., 2019). It has also been reported that overexpression of the stilbene synthase (STS) gene, a key gene for resveratrol synthesis, in plants can result in resveratrol synthesis in transgenic plants (Hain et al., 1990; Hain et al., 1993; Rimando et al., 2012). On the other hand, although the production of pinosylvin has been reported in engineered bacteria and yeast (van Summeren-Wesenhagen and Marienhagen, 2015), there are no reports on the production of pinosylvin and its methylated derivatives by introducing the STS gene into plants. For example, overexpression of the *Pinus sylvestris STS* gene in poplar did not result in the synthesis of pinosylvin stilbenes (Seppänen et al., 2004). Seppänen et al. (2004) suggested that the failure of stilbene synthesis by heterologous expression of the *P. sylvestris STS* gene in poplar may occur by restriction of the cinnamoyl CoA supply or metabolic channeling.

Resveratrol (3,4’,5-trihydroxystilbene) and pinosylvin (3,5-dihydroxystilbene) are very similar in structure. Although the key enzymes involved in pinosylvin and resveratrol synthesis are functionally homologous (Tropf et al., 1995; Dubrovina and Kiselev, 2017), the precursors involved in their synthesis pathways are different: pinosylvin is synthesized from phenylalanine, cinnamic acid, and cinnamoyl-CoA while resveratrol is synthesized from tyrosine, coumaric acid, and coumaroyl-CoA (Valletta et al., 2021).

In pine species, pinosylvin and dihydropinosylvin are present as intermediates, with mostly methylated pinosylvin derivatives occurring at relatively high concentrations. In *P. strobus*, two substances accumulate as the main methylated pinosylvin derivatives, dihydropinosylvin monomethyl ether (DPME) and pinosylvin monomethyl ether (PME), and the proportion of DPME is much greater than that of PME (Hwang et al., 2021). On the other hand, in *Pinus densiflora*, *Pinus koraiensis*, and *Pinus sylvestris*, PME is present in a much greater percentage than DPME (Dumas et al., 1983; Ekeberg et al., 2006; Kim et al., 2022). In *P. rigida*, pinosylvin dimethyl ether is present at the highest concentration in branches and leaves even under unstressed conditions (Hwang et al., 2022).

Pine wilt disease (PWD) caused by PWN infection is the most serious disease worldwide, and its control is challenging (Hirata et al., 2017). In many countries, many native pine species in Asia and Europe are severely damaged by PWNs, and the extent of PWD is increasing annually (Mamiya, 1983; Futai, 2013; Hirata et al., 2017). Pinosylvin and its methylated derivatives (pinosylvin, PME, and DPME) found in pine tree species are reported to be highly toxic to PWNs (Suga et al., 1993; Hwang et al., 2021). Therefore, the ability of pine tree species to synthesize pinosylvin stilbenes after infection with PWNs may appear to be related to resistance to PWD.

All pine species possess STS and PMT enzymes, key enzymes involved in the synthesis of pinosylvin and its methylated derivatives (Gehlert et al., 1990; Chiron et al., 2000). In *P. strobus*, the synthesis of pinosylvin derivatives (PME and DPME) increased significantly upon infection with PWNs, whereas *P. densiflora* and *P. koraiensis* showed no change in the synthesis of pinosylvin derivatives upon PWN infection (Hwang et al., 2021). This finding suggested that the regulation of the genes involved in the synthesis of pinosylvin derivatives is differs between PWN-resistant and PWN-susceptible pine species.

In general, transcription factors (TFs) are known to regulate gene expression by binding to the DNA (promoter) of a specific gene, where they act alone or in combination with other proteins (Liu et al., 1999). The involvement of TFs in resveratrol synthesis in *Vitis* plants has been studied in detail. In *Vitis vinifera*, R2R3-MYB TFs (*MYB14* and *MYB15* genes) regulate resveratrol biosynthesis (Höll et al., 2013). In addition, the WRKY family was also identified as the main TF regulating resveratrol biosynthesis in *Vitis* (Vannozzi et al., 2018). WRKY TFs interact with MYB TFs to control resveratrol biosynthesis in grapevine (Jiang et al., 2019; Mu et al., 2023). However, the TFs involved in the synthesis of pinosylvin stilbene biosynthesis have not been characterized in pine species. In a previous study, we selected several TFs by transcriptome analysis after the inoculation of PWNs in *P. strobus*, and a gene (*PsbHLH1*) encoding a bHLH-type TF exhibited the strongest expression upon PWN infection (Hwang et al., 2021). Moreover, the expression of the *PsbHLH1* gene and the timing of increased pinosylvin stilbene synthesis were strongly correlated.

*P. strobus* pine is resistant to PWD, so there is no need to develop transgenic plants through genetic transformation. However, Korean red pine and Korean pine are highly susceptible to PWD, so it is worth developing PWD-resistant pine through genetic transformation. Therefore, in this study, experiments were conducted to introduce the *PsbHLH1*, *PsSTS*, and *PsPMT* genes into cultured PWN-susceptible Korean pine cells and to investigate whether there was an increase in PME and DPME in these transgenic lines. We confirmed that the overexpression of a bHLH-type TF (*PsbHLH1*) in *P. strobus* is an important gene for the production of PME and DPME via heterologous expression in transgenic cells of *P. koraiensis* and transgenic tobacco leaves.

## Materials and methods

### Sequence analysis of the *PsbHLH1* gene

The deduced amino acid sequence of PsbHLH1 was analyzed via the Simple Modular Architecture Research Tool (SMART) (Schultz et al., 1998). Multiple alignment of bHLH amino acid sequences was performed by ClustalW (Higgins et al., 1994). A phylogenetic tree was constructed by the neighbor-joining method using a bootstrap test with 1,000 replications in Phylip (http://evolution.gs.washington.edu/phylip.html). The phylogenetic tree was visualized in MEGA5 (Tamura et al., 2011). The analysis of the domain architecture of the PsbHLH1 protein was performed by SMART (Letunic et al., 2021). The TF binding motifs in the promoter and 5′ untranslated (UTR) regions of phenylalanine ammonia-lyase (PAL), 4-coumarate-CoA ligase (4CL), pinosylvin synthase (STS), pinosylvin *O*-methyltransferase (PMT), and acetyl-CoA carboxylase (ACC) in Korean pine (*Pinus koraiensis*) or other pine species were analyzed using the Softberry NSITE Program (version 5.2013, http://www.softberry.com).

### Construction of binary vectors

To isolate the genes encoding pinosylvin synthase (*PsSTS*), pinosylvin methyltransferase (*PsPMT*), and TF (*PsbHLH1*) in *P. strobus*, mRNA was extracted from cultured cells using the RNeasy® Plant Mini Kit (Qiagen, Germany) and cDNA was prepared using reverse transcriptase. The primers used to obtain the full-length cDNA of these three genes were prepared as shown in **Supplementary Table S1**, and then the cDNA was subsequently amplified via PCR using the template to obtain the full-length cDNA.

A total of three binary vectors were constructed: a vector for the *PsbHLH1* gene; a vector containing two genes, *PsSTS* and *PsPMT*, for coexpression; and a vector containing three genes, *PsSTS*, *PsPMT*, and *PsbHLH1*, for coexpression. The first vector used the hygromycin phosphotransferase (*HPT*) gene as the selection marker, and the second and third vectors used the Basta-resistant gene (*BAR*) as the selection marker. These three binary vectors were mobilized into *Agrobacterium tumefaciens* GV3101 for plant transformation.

### Generation of transgenic calli of Korean pine

To obtain transgenic calli, mature zygotes of Korean pine (*Pinus koraiensis*) seeds were used. Mature zygotes were isolated from seeds following the methods of Kim et al. (2022). The culture conditions for inducing calli from the culture of zygotic embryos of *P. koraiensis* were the same as those in Kim et al. (2022). Mature zygotic embryos were soaked in *Agrobacterium* suspended in 1/2 LV medium (Litvay et al., 1985) supplemented with 50 μg/ml acetosyringone for 10 min and then subsequently placed on sterilized filter paper. After approximately 1 h, the zygotic embryos were transferred to 1/2 LV medium supplemented with 0.5 mg/L 2,4-D and 0.5 mg/L BA and cocultured for 3 days. The embryos were then transferred to the same medium supplemented with 500 mg/L cefotaxime and cultured for 2 weeks, followed by 2 passages at 2-week intervals to eliminate *Agrobacterium*. Transgenic calli induced from zygotic embryos were then selected by transfer to medium supplemented with 10 mg/L hygromycin and subculturing on the same medium at 2-week intervals. When a hygromycin-resistant callus was obtained, a callus from each zygotic embryo line was selected as an independent line. The same protocol for the construction of transgenic calli was used for the other two vectors containing the *BAR* gene marker, and 20 mg/L Basta was added for the selection of transgenic calli.

### PCR analysis

Genomic polymerase chain reaction (PCR), reverse transcription PCR (RT-PCR), and reverse transcription quantitative PCR (RT-qPCR) were performed to investigate the introduction and expression of the genes in the transgenic pine callus and transgenic tobacco plants. After the cultured cells were transformed with the pine *PsbHLH1* gene, genomic DNA was isolated from the transgenic calli using the Genomic DNA Extraction Kit Mini (RBC bioscience, New Taipei City, Taiwan). Genomic PCR was performed using *PsbHLH1* and *HPT* gene primers as shown in **Table S1**. The selected lines were subjected to RT-PCR and RT-qPCR using cDNA obtained from the transgenic lines as a template to investigate the expression of the introduced genes. The RT-PCR and RT-qPCR information for the amplification of the *PsbHLH1* and *HPT* genes are shown in **Table S1**. For RT-qPCR, the pine *β-actin* gene was used as a reference gene.

### Transient expression of genes in tobacco leaves by infiltration

**Figure 1:**
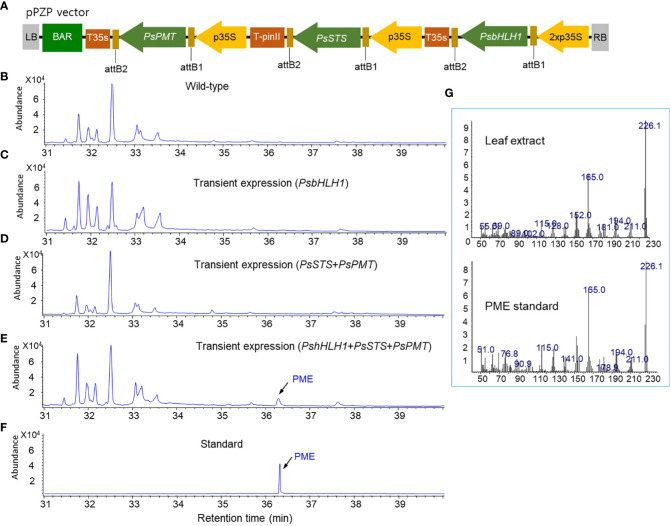
Transient expression of the vectors by agroinfiltration to production PME in tobacco leaves. **(A)** A pPZP binary vector for the overexpression of *PsbHLH1, PsSTS*, and *PsPMT* genes. **(B)** GC chromatogram of a wild-type tobacco leaf. **(C)** GC chromatograms of tobacco leaves transiently expressing *PsbHLH1* gene after two days of agroinfiltration. **(D)** GC chromatograms of tobacco leaves following transient expression of the *PsSTS* and *PsPMT* genes after two days of agroinfiltration. **(E)** GC chromatogram of tobacco leaves following transient expression of the *PsSTS*, *PsPMT*, and *PsbHLH1* genes after two days of agroinfiltration. **(F)** GC chromatograms of standard DPME and PME. **(G)** Mass fraction of the PME peak produced in tobacco leaves by agroinfiltration and the standard PME peak.

*A. tumefaciens* harboring two binary vectors, as shown in **Figures 1A, B**, was harvested and prepared for infiltration. The bacterial suspension was infiltrated into the intercellular spaces of the leaf tissue of tobacco (*Nicotiana tabacum* cv Xanthi) using a needleless syringe. After infiltration, the infiltrated tobacco plants were usually kept in a growth chamber for 2 days. The infiltrated leaf tissues were harvested and subjected to analysis for the production of pinosylvin stilbenes.

### Analysis of pinosylvin stilbenes by GC/MS

Calli from the untransformed control and the three transgenic lines were sampled for GC analysis, then dried in a 50°C dry oven and ground into powder. One gram of each powder was placed in 100% MeOH, sonicated for 30 min at 40°C, and centrifuged at 15000 ×g. The supernatant was filtered through a membrane filter. For GC analysis of tobacco leaf tissues, control and infiltrated tobacco leaves were dried in a 50°C dry oven and ground into powder. One hundred milligrams of each powder were placed in 100% MeOH, sonicated for 30 min at 40°C, and centrifuged at 15000 ×g, after which the supernatant was filtered through a membrane filter.

The filtered aliquots were analyzed by GC (Agilent 7890A) with an Agilent 5975C MSD system equipped with an HP-5MS capillary column (30 m × 0.25 mm, film thickness 0.25 mm). The injection temperature was 250°C. The column temperature was as follows: 70°C for 4 min, 220°C at a rate of 5°C min^-1^, heating at 4°C min^-1^ up to 320°C, and holding at 320°C for 5 min. The carrier gas was He. The interface temperature was 300°C, and split/splitless injection (10:1) was used. The ionization chamber temperature was 250°C, and ionization was performed by electron impact at 70 eV. The standards of PME and DPME used in GC-MS analysis were purchased from Sigma–Aldrich Co. The experiment was performed in triplicate and repeated three times.

### Statistical analysis

Quantitative data are expressed as the mean values ± standard error (SE). All the statistical analysis were performed with SPSS software (SPSS Science, Chicago, IL, USA). Statistical differences among means were calculated using one-way ANOVA followed by Duncan’s *post hoc* analysis.

## Results

### Isolation and expression of *PsbHLH1*

**Figure 2:**
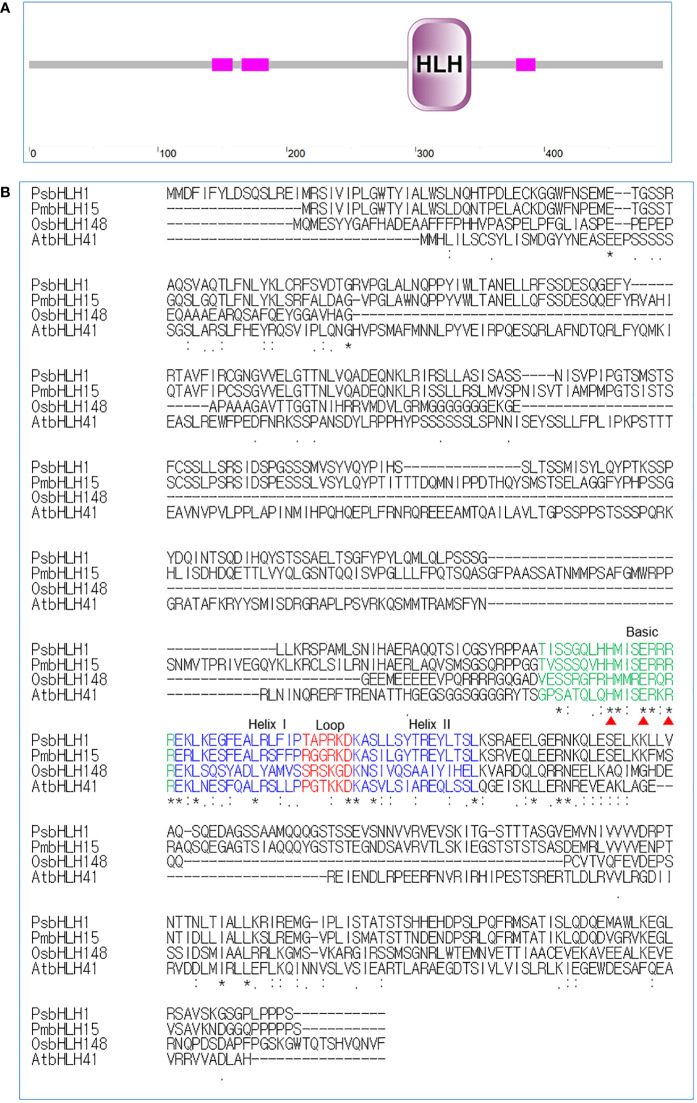
Sequence analysis and multiple alignments of the bHLH domains in the bHLH1 proteins of *Pinus strobus* and those of other species. **(A)** Protein domain analysis of PsbHLH1 protein using SMART. **(B)** Conserved amino acid analysis of bHLH domains in the PsbHLH1 protein of *Pinus strobus*, bHLH15 protein of *Pinus massoniana* (UFA45712.1), bHLH148 protein of *Oryza sativa* (NP_001389178.1), and bHLH41 protein of *Arabidopsis thaliana* (At5G56960). Amino acid residues that induce specific DNA recognition by MYC2 are labelled with red triangles.

The *PsbHLH1* gene was cloned from *P. strobus* and deposited in the NCBI GenBank database under accession number GIIE01106737.1. The open reading frame (ORF) of *PsbHLH1* is 1479 bp in length and encodes a bHLH family protein of 492 amino acids, with a calculated molecular weight of 54.31 kDa. BLAST analysis revealed that PsbHLH1 shares the highest identity with the functionally unknown *Pinus massoniana* bHLH15 and 55% identity with the *Arabidopsis thaliana* AtbHLH041, which belongs to the group IVd subfamily. Domain architecture analysis via SMART (Letunic et al., 2021) revealed that the PsbHLH1 protein belongs to the MYC-type bHLH domain (**Figure 2A**). The bHLH domain comprises two alpha helices connected by a nonconserved loop region and is used for dimerization, and the basic domain is used for DNA binding (Jones, 2004). Multiple sequence alignment revealed that PsbHLH1 contains a conserved bHLH domain that has four conserved regions, namely, the basic region, two helixes, and a loop region (**Figure 2B**).

Since the genomic sequence of *Pinus strobus* has not been published, we analyzed the promoters of the *PAL*, *4CL*, *STS*, *PME*, and *ACC* genes in the *Pinus lambertiana* whole-genome shotgun sequence (GenBank: LMTP000000000.1) using NSITE-PL (version 5.2013, at http://www.softberry.com). The promoter regions of the *PAL*, *4CL*, *STS*, and *PME* genes in *Pinus lambertiana* lacked a G-box motif for MYC-type bHLH protein binding. However, only the *ACC* gene had a MYC2 binding G-BOX motif (CACGTG) in the 5-UTR, not in the promoter region of the *ACC* gene. The regulatory elements in the untranslated regions (UTRs) of the *ACC* genes of all 5 selected *Pinus* species (*P. koraiensis*, *P. strobus, Pinus sylvestris, Pinus taeda*, and *Pinus flexilis*) were also examined. All of these genes had a MYC2-binding G-BOX motif (CACGTG) in the 5’ -UTR (**Supplementary Figure S1**).

**Figure 3:**
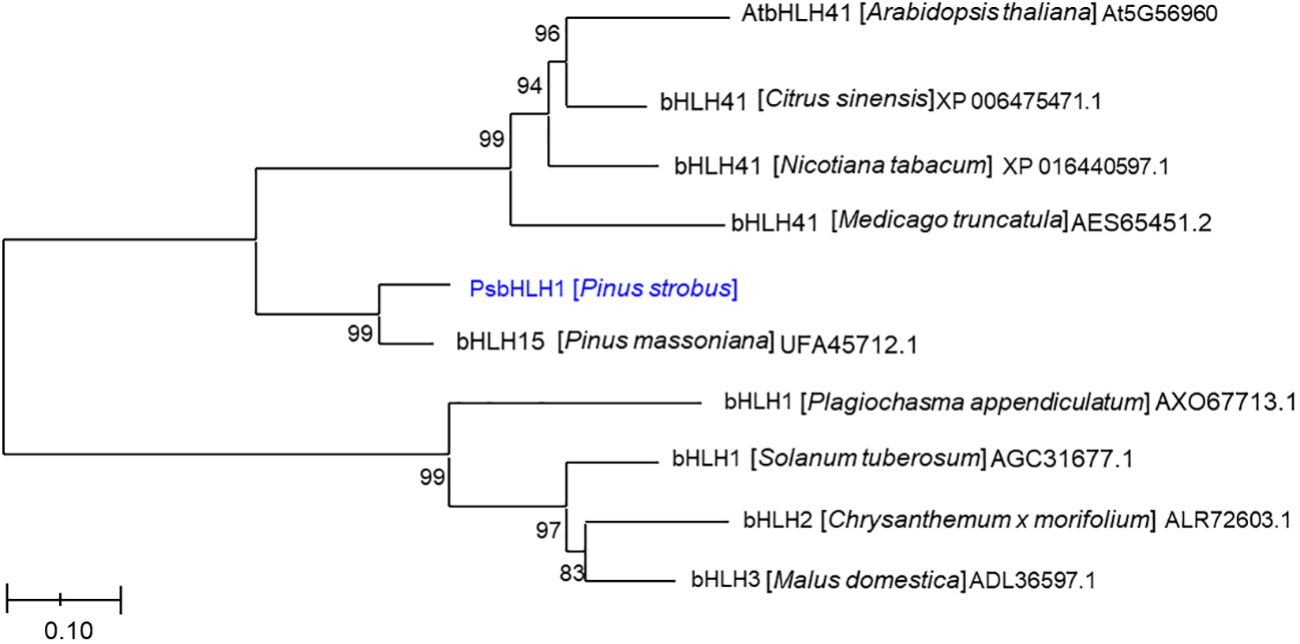
Phylogenetic analysis of the PsbHLH1 protein among the proteins of other plant species by Neighbor-joining (NJ) phylogenetic analysis using the MEGA 6.0 software program. The scale bar represents 0.05 amino acid substitutions per site.

Phylogenetic analysis based on the neighbor-joining algorithm revealed that PsbHLH1 was first clustered with a functionally unknown *bHLH15* gene of *P. massoniana* and was also related to a subgroup (IVd) that included the functionally unknown *AtbHLH041* gene (**Figure 3**) (Heim et al., 2003). AtbHLH041 is a transcriptional repressor of root stem cell factors during the establishment of auxin-induced callus pluripotency (Xu et al., 2024). However, PsbHLH1 is an independent group in which *Plagiochasma appendiculatum* bHLH1 protein regulates bis-bibenzyl synthesis (Wu et al., 2018), *Solanum tuberosum* bHLH1 protein regulates phenylpropanoid metabolism (Payyavula et al., 2013), *Chrysanthemum x morifolium* bHLH2 is involved in anthocyanin biosynthesis (Xiang et al., 2015), and *Malus domestica* bHLH3 activates anthocyanin biosynthesis (Xie et al., 2017).

### Enhanced production of PME and DPME in transgenic calli of *P. koraiensis* overexpressing *PsbHLH1*

**Figure 4:**
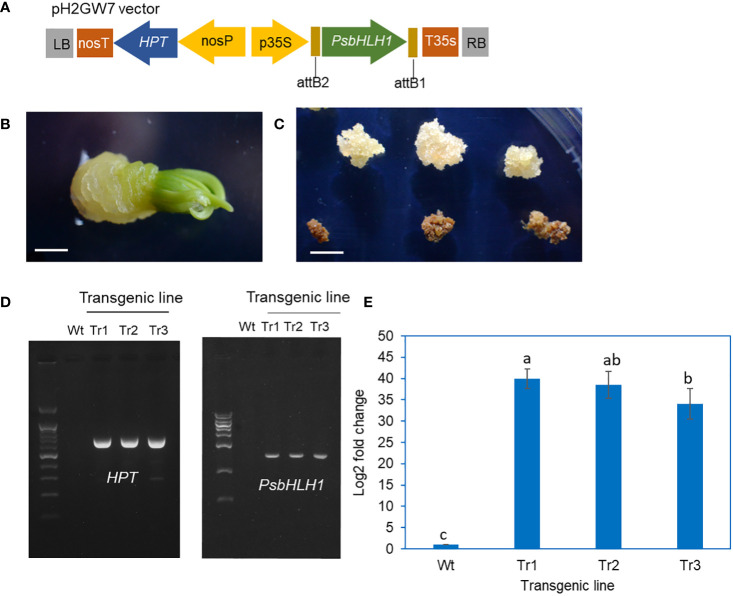
Construction of transgenic *P. koraiensis* calli overexpressing the *PsbHLH1* gene. **(A)** A pH2GW7 binary vector for overexpression of the *PsbHLH1* gene. **(B)** Callus induction of *P. koraiensis* zygotic embryos cultured on 1/2 LV medium with 500 mg/L cefotaxime. The scale bar is 3 mm. **(C)** Putative transgenic callus cultured on 1/2 LV medium with 500 mg/L cefotaxime and 20 mg/L hygromycin. The scale bar is 5 mm. Calluses positioned on the upper side survived on medium with 20 mg/L hygromycin. **(D)** Genomic PCR of genes (*HPT* and *PsbHLH1*) in wild-type and transgenic callus lines. **(E)** RT-qPCR analysis of the *PsbHLH1* gene in wild-type and transgenic *P. koraiensis* callus lines overexpressing the *PsbHLH1* gene.

A vector for overexpressing of the *PsbHLH1* gene with the hygromycin selection marker gene (*HPT*) was constructed for the transformation of *P. koraiensis* (**Figure 4A**). Hygromycin-resistant calli were obtained by the subculture of calli derived from zygotic embryos after cocultivation with *Agrobacterium* harboring the *PsbHLH1* gene (**Figures 4B, C**). The final selection of transgenic callus lines was obtained after more than five times subcultures on a hygromycin-containing medium. Three hygromycin-resistant calli were selected from different zygotic lines.

To confirm the gene introduction in these transgenic lines, primers for the *HPT* and *PsbHLH1* genes were prepared, and PCR was performed on genomic DNA from the control untransformed callus and transgenic lines. The *PsbHLH1* gene and the selective marker *HPT* gene were not amplified from the control, but in the transgenic line, the *HPT* and *PsbHLH1* genes were amplified (**Figure 4D**). We also extracted mRNA from the calli of the control and transgenic lines and performed RT-qPCR using cDNA as a template; the results revealed that the expression level of the *PsbHLH1* gene was strongly increased in all three transgenic lines (**Figure 4E**).

**Figure 5:**
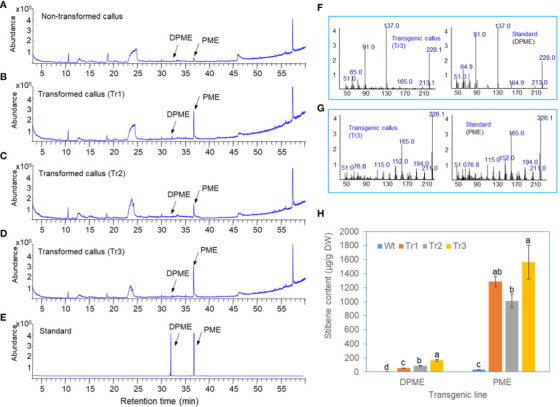
DPME and PME analysis of wild-type and transgenic calli of *P. koraiensis* overexpressing PsbHLH1. **(A)** GC chromatogram of wild-type calli. **(B-D)** GC chromatograms of the transgenic callus lines. **(E)** GC chromatograms of standard DPME and PME. **(F)** Mass fraction of a peak in a transgenic callus and standard DPME compound. **(G)** Mass fraction of a peak in a transgenic callus (Tr3) and standard PME compound. **(H)** Contents of DPEM and PME in wild-type and transgenic callus lines. Different letters above the bars indicate statistically significant differences at P<0.05 (one-way ANOVA).

Three lines of transgenic calli overexpressing *PsbHLH1* and wild-type control calli were analyzed via GC/MS. In the control, untransformed callus, a small PME peak was detected at a retention time of 36.8 min, and a very small peak of DPME was identified at a retention time of 32 min (**Figure 5A**). The calli of the transgenic lines were characterized by clearly enhanced PME and DPME peaks in the GC chromatogram (**Figures 5B–D**), and quantification of these two substances revealed a more than 32-fold increase in PME and a 10-fold increase in DPME compared to those in the control (**Figure 5H**). The two substances were identified by comparing the retention times of the DPME and PME standards (**Figure 5E**) and by comparing the mass fraction patterns of these two substances (**Figures 5F, G**).

**Figure 6:**
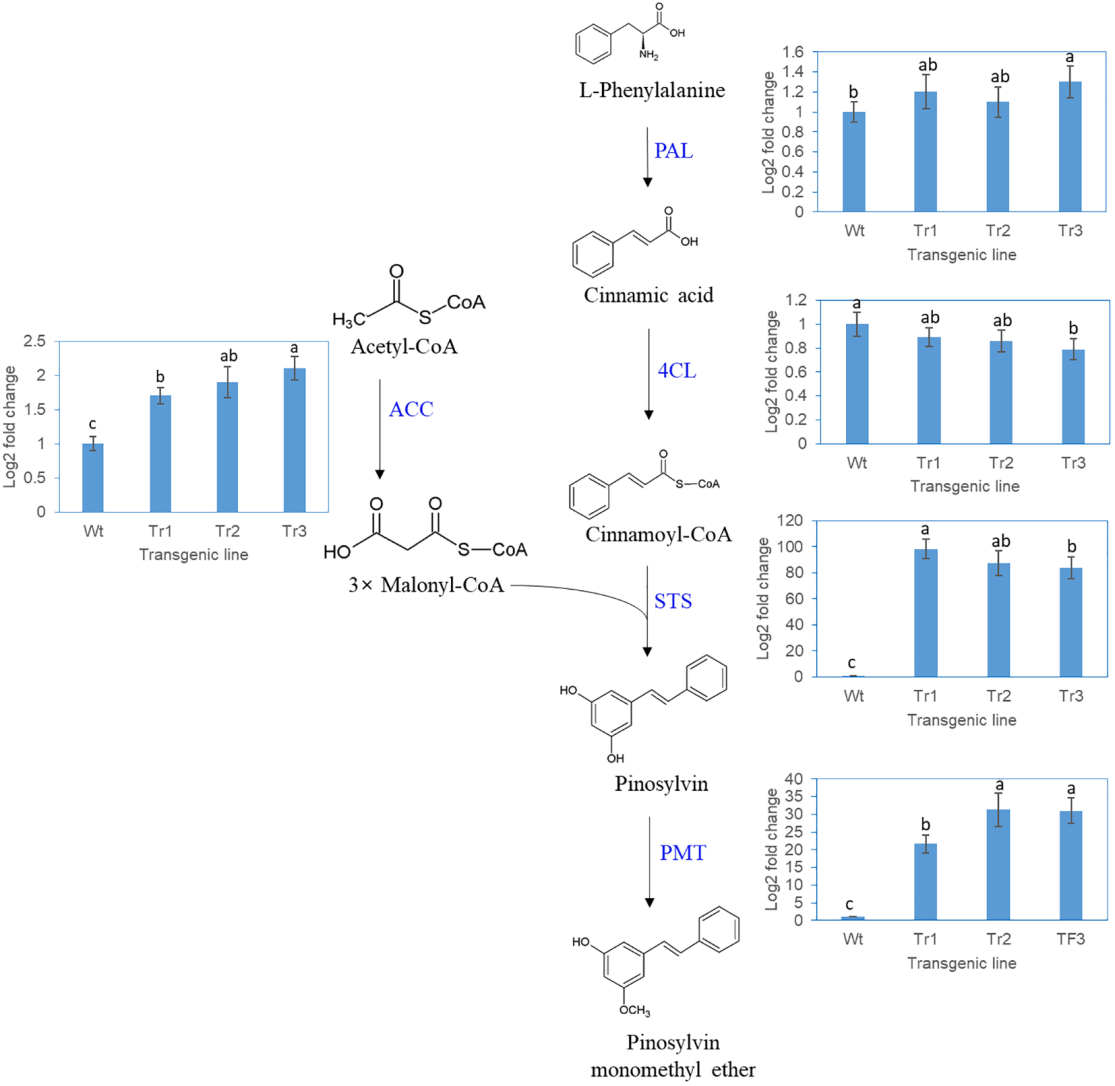
RT-qPCR analysis of *P. koraiensis* genes involved in pinosylvin monomethyl ether biosynthesis in wild-type and transgenic calli of *P. koraiensis* overexpressing *PsbHLH1*. PAL, phenylalanine ammonia-lyase; 4CL, 4-coumarate-CoA ligase; STS, pinosylvin synthase; PMT, pinosylvin *O*-methyltransferase; ACC, acetyl-CoA carboxylase. Different letters above the bars indicate statistically significant differences at P<0.05 (one-way ANOVA).

The genes in the PME and DPME biosynthesis pathway included the *PAL*, *4CL*, *STS, PME*, and *ACC* genes, as shown in **Figure 6**. Pinosylvin is produced by combining one molecule of the cinnamoyl-CoA gene and three molecules of malonyl-CoA, and the *ACC* gene is involved in the synthesis of malonyl-CoA. The *STS* gene makes the backbone of pinosylvin, and the *PMT* gene attaches the methyl group to pinosylvin. In a transgenic *P. koraiensis* callus line (line 3) in which the *PsbHLH1* gene was overexpressed, the expression of the *PAL* and *4CL* genes was unchanged in the transgenic line compared to the control (**Figure 6**). On the other hand, the *STS* and *PMT* genes of *P. koraiensis* were more highly expressed in the transgenic lines than in the control. In addition, the expression of the *ACC* gene involved in malonyl-CoA synthesis in of *P. koraiensis* was significantly greater than that in the control (**Figure 6**).

### DPME and PME accumulation in transgenic calli of *P. koraiensis* overexpressing *PsSTS* and *PsPMT* genes

**Figure 7:**
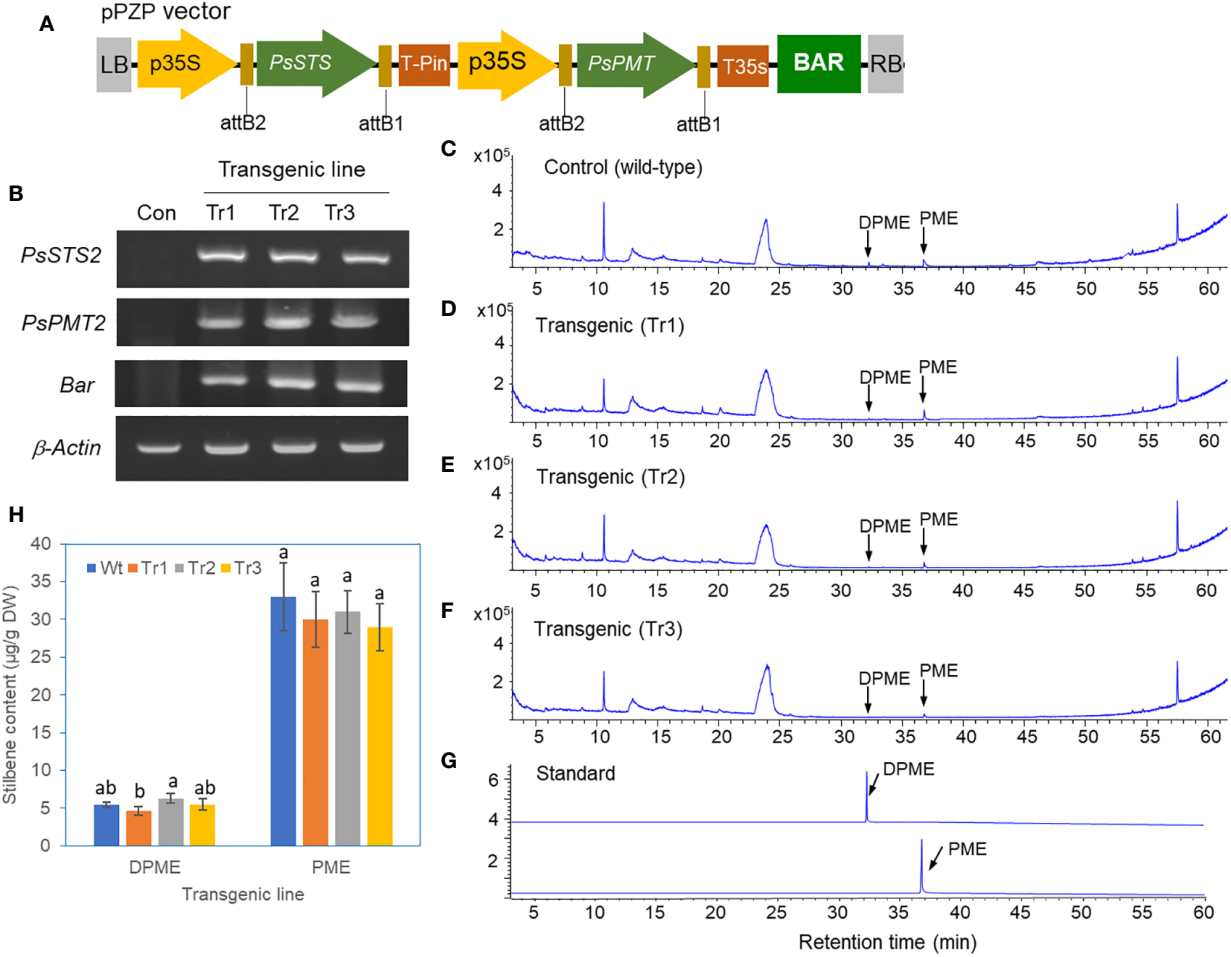
Analysis of DPME and PME in transgenic *P. koraiensis* calli overexpressing the *PsSTS* and *PsPMT* genes. **(A)** A pPZP binary vector for the overexpression of both the *PsSTS* and *PsPMT* genes. **(B)** Expression of the *PsSTS* and *PsPMT* genes in wild-type and transgenic *P. koraiensis* callus lines determined by RT-PCR. **(C)** GC chromatograms of DPME and PME in wild-type calli. **(D-F)** GC chromatograms of DPME and PME in three transgenic lines. **(G)** GC chromatograms of standard DPME and PME. **(H)** Contents of DPME and PME in the wild-type and transgenic callus lines. Different letters above the bars indicate statistically significant differences at P<0.05 (one-way ANOVA).

A vector for the overexpression of both the *PsSTS* and *PsPMT* genes with the *BAR* selection marker gene (*BAR*) was constructed for the transformation of *P. koraiensis* (**Figure 7A**). Basta-resistant calli were obtained using a protocol similar to that used for *PsbHLH1* overexpression. The final selection of three Basta-resistant callus lines was confirmed by RT-PCR of the introduced genes. The expression of the *PsSTS* and *PsPMT* genes in *P. koraiensis* calli was detected in all three transgenic lines but not in the wild-type control (**Figure 7B**).

Three lines of transgenic calli lines overexpressing the *PsSTS* and *PsPMT* genes and the wild-type control were analyzed via GC-MS. The PME and DPME peaks in the GC chromatogram were not different between the transgenic lines and the untransformed control (**Figures 7C–G**), and quantification of these two substances revealed no significant difference between them (**Figure 7H**).

### Transient expression of PME by transient expression of *PsbHLH1*, *PsSTS*, and *PsPMT* in tobacco leaves resulted in the production of PME

To analyze the production of PME and DPME in transgenic tobacco leaves by transient expression, the two vectors used are described above (**Figures 4A**, **7A**), and the vector coexpressing three genes, *PsbHLH1*, *PsSTS*, and *PsPMT* (**Figure 1A**), was used. Tobacco leaves were infiltrated with *Agrobacterium* solution using a transient expression system with these two vectors, after which then the leaves were dried and analyzed via GC/MS after 2 days. No pinosylvin derivatives (PME or DPME) were detected in wild-type tobacco leaves (**Figure 1B**). In the transient expression of *PsbHLH1* (**Figure 1C**), or *PsSTS* and *PsPMT* genes (**Figure 1D**), no pinosylvin derivatives (PME or DPME) were detected. On the other hand, transient expression of the three *PsbHLH1*, *PsSTS*, and *PsPMT* genes in tobacco leaves resulted in the production of PME (**Figure 1E**), which was confirmed by comparing the retention time (**Figure 1F**) and mass fraction pattern (**Figure 1G**) of the standard PME compound.

## Discussion

### Overexpression of *PsbHLH1* in transgenic *P. koraiensis* calli resulted in the production of pinosylvin stilbenes

Transcriptome analysis following PWN infection of *Pinus strobus* revealed that the *PsbHLH1* TF gene had the highest expression value upon PWN infection among all the TF genes analyzed, and RT-qPCR analysis after PWN infection also revealed *PsbHLH1* as the most promising TF for responding to PWN infection (Hwang et al., 2021). In the present study, we investigated whether this gene regulates the biosynthesis of pinosylvin stilbenes in PWN-susceptible Korean pine (*P. koraiensis*). In transgenic *P. koraiensis* calli overexpressing the *PsbHLH1* gene, the production of PME and DPME significantly increased. This result suggested that the *PsbHLH1* gene is an important TF for the regulation of pinosylvin stilbene biosynthesis. To analyze whether the overexpression of the *PsbHLH1* gene affects the genes involved in pinosylvin biosynthesis in *P. koraiensis*, the expression of *P. koraiensis* genes in transgenic *P. koraiensis* callus lines was analyzed via RT-qPCR. The analysis revealed that the expression of the *Pk*ACC gene of *P. koraiensis*, which is involved in the synthesis of malonyl-CoA, an important precursor in the synthesis of pinosylvin, was significantly increased. Moreover, the expression of the *PkSTS* and *PkPMT* genes of *P. koraiensis* significantly increased, while the expression of the *PkPAL* and *Pk4CL* genes of *P. koraiensis* did not change. These results suggest that the ectopic expression of the *PsbHLH1* gene in transgenic *P. koraiensis* calli affects the expression of the *Pk*ACC, *PkSTS*, and *PkPMT* genes, which results in increased production of pinosylvin derivatives in *P. koraiensis* calli.

The MYC2 bHLH TF is known to be a master regulator that activates defense gene expression and responds to JA and has been reported to be involved in the regulation of specialized metabolites (Du et al., 2017; López-Vidriero et al., 2021; Luo et al., 2023). PsbHLH1 is a MYC-type bHLH. PsbHLH1 shares the highest identity with *Arabidopsis thaliana* AtbHLH041, which belongs to the group IVd subfamily. The role of subgroup IVd within the bHLH TF family in the biosynthesis of secondary compounds has not been characterized.

We analysed the promoter and UTR regions of the *PAL*, *4CL*, *STS*, *PME*, and *ACC* genes in *Pinus lambertiana* and found that the promoter regions of the *PAL*, *4CL*, *STS*, and *PME* genes lacked a bHLH binding domain. On the other hand, the *ACC* gene has a bHLH binding G-box motif (CACGTG) in the 5-UTR. This G-box motif was commonly present in the UTRs of all five *Pinus* species (**Supplementary Figure S1**). MYC-type bHLH TFs bind to the G-box (5′-CACGTG-3′), a DNA motif typically recognized by bHLH proteins from plants, yeast, and animals (López-Vidriero et al., 2021). This result suggested that the PsbHLH1 TF may bind to the UTR of the *ACC* gene and regulate its expression. ACC is an enzyme that catalyzes the conversion of acetyl-CoA to malonyl-CoA which is an essential building block for the biosynthesis of fatty acids, polyketides, stilbenoids, and flavonoids (Wu et al., 2017). Indeed, malonyl-CoA is an important precursor in the synthesis of resveratrol stilbene (Xu et al., 2011; Yang et al., 2015). In the metabolic engineering of *Escherichia coli* for the synthesis of the pinosylvin stilbene, malonyl-CoA availability in the heterologous host is thought to be the main bottleneck for pinosylvin production (van Summeren-Wesenhagen and Marienhagen, 2015).

No information is available on the transcriptional regulators involved in pinosylvin stilbene synthesis in plant species. There is some information on the TFs involved in resveratrol synthesis in grapes. Two R2R3-type MYB genes, MYB14 and MYB15, have been identified as transcriptional regulators of resveratrol stilbene biosynthesis in *Vitis vinifera* (Höll et al., 2013). WRKY (VviWRKY03) and MYB TF (VviMYB14) in *Vitis vinifera* have been reported to combinatorically regulate the stilbene synthase gene (Vannozzi et al., 2018). In particular, VviWRKY24 seems to act as a singular effector in the activation of the stilbene synthase (STS) promoter, while VviWRKY03 acts through a combinatorial effect with VviMYB14 (Vannozzi et al., 2018). Overexpression of VaMyb40 and VaMyb60 resulted in stilbene biosynthesis in cell cultures of *Vitis amurensis* (Ananev et al., 2022). Although the STS genes for pinosylvin synthesis in pine plants and resveratrol synthesis in dicots are holomorphic, they utilize different precursors to synthesize the two stilbenes. Pinosylvin stilbene is synthesized by the coupling of one molecule of cinnamoyl-CoA and three molecules of malonyl-CoA, whereas resveratrol is synthesized by the coupling of coumaroyl-CoA and malonyl-CoA (Valletta et al., 2021).

### Overexpression of key genes (*PsSTS* and *PsPMT*) involved in pinosylvin biosynthesis in transgenic *P. koraiensis* calli did not change the production of pinosylvin stilbene

In transgenic *P. koraiensis* calli overexpressing the *PsSTS* and *PsPMT* genes, the production of PME and DPME in transgenic calli was unaffected. Similarly, the overexpression of the *Pinus sylvestris STS* gene in poplar did not result in the synthesis of pinosylvin or its derivatives (Seppänen et al., 2004). Malonyl-CoA is a key precursor of pinosylvin bioproduction (Liang et al., 2016). In the metabolic engineering of *E. coli*, malonyl-CoA availability is thought to be the main bottleneck for pinosylvin production (van Summeren-Wesenhagen and Marienhagen, 2015). ACC is an important enzyme that catalyzes the production of malonyl-CoA (Wu et al., 2017). Interestingly, the PsbHLH1 TF may bind to the MYC2-binding G-box motif (CACGTG) in the UTR of the *ACC* gene. Thus, a transcriptional regulator, PsbHLH1, may be essentially needed for the malonyl-CoA precursor supply in the biosynthesis of pinosylvin stilbenes. Our study is the first to report the production of pinosylvin stilbenes by heterologous expression of genes in plants.

### The production of pinosylvin derivatives in tobacco leaves requires the *PsbHLH1* gene

In our experiment, the transient expression of the *PsSTS* and *PsPMT* genes in tobacco resulted in no production of pinosylvin stilbenes. The same result was obtained for the transient expression of the *PsbHLH1* in tobacco. Like in our study, no pinosylvin stilbene was produced when the *Pinus sylvestris STS* gene was overexpressed in poplar (Seppänen et al., 2004). Seppänen et al. (2004) suggested that the failure of stilbene synthesis by heterologous expression of the *P. sylvestris STS* gene in poplar may occur by restriction of the cinnamoyl-CoA supply or metabolic channeling. Moreover, the overexpression of pinosylvin synthase genes (*PjSTS1a*, *PjSTS2*, or *PjSTS3*) derived from the coniferous tree (*Picea jezoensis*) in transgenic calli of grape plants resulted in the production of resveratrol-related stilbenes but not pinosylvin stilbenes (Suprun et al., 2020). Tobacco plants do not produce stilbenes such as pinosylvin or resveratrol, and may not harbor stilbene synthase genes. Thus, the lack of pinosylvin stilbenes during transient expression of *PsbHLH1* in tobacco may be expected. However, in our study, coexpression of the *PsbHLH1* gene together with the *PsSTS* and *PsPMT* genes in tobacco leaves resulted in the production of pinosylvin derivatives. The requirement of the *PsbHLH1* gene for the production of PME in tobacco leaves by transient expression indicated that PsbHLH1 may play an important regulatory role in the biosynthesis of pinosylvin stilbenes. Analysis of the molecular mechanism of the PsbHLH1 protein may shed light on its regulatory role in PME and DPME biosynthesis.

In conclusion, although the two genes, *PsSTS* and *PsPMT*, are known to be key players in pinosylvin stilbene biosynthesis, the overexpression of these two genes did not result in either PME in either *P. koraiensis* callus or tobacco leaves, but additional expression of *PsbHLH1* successfully induced pinosylvin stilbene production. These results indicate that *PsbHLH1* is an important TF for pinosylvin stilbene biosynthesis, and our study is the first to report the production of pinosylvin stilbene using plant genetic engineering technology. These findings suggest that introducing the *PsbHLH1* gene into PWN-susceptible pine species will increase the amount of pinosylvin stilbene compounds, which are highly toxic to PWN, and thus increase its resistance to PWN.

## Data availability statement

The datasets presented in this study can be found in online repositories. The names of the repository/repositories and accession number(s) can be found below: https://www.ncbi.nlm.nih.gov/genbank/, GIIE01106737.1.

## Author contributions

YK: Data curation, Formal analysis, Investigation, Writing – review & editing. JH: Data curation, Formal Analysis, Investigation, Writing – review & editing. YC: Conceptualization, Funding acquisition, Supervision, Writing – original draft.
