# Supplementary material for: A Pinus strobus transcription factor PsbHLH1 activates the production of pinosylvin stilbenoids in transgenic Pinus koraiensis calli and tobacco leaves
Source: Front Plant Sci. 2024 Jan 18;15:1342626. doi: 10.3389/fpls.2024.1342626 (PMC10830828; doi:10.3389/fpls.2024.1342626)
Supplement: Supplementary file 1 [file Table_1.docx]

| Gene  (for experiment) | Primer | |
| --- | --- | --- |
|  | Forward primer | Reverse primer |
| PkPAL  (qPCR) | AGAACGCAGACGGTGAGAAG | CTGGCCAGATTGACTTGCAGTT |
| Pk4CL  (qPCR) | CCTATGCCGCCTGTGTAGAA | GGACAGTACTCGCTCTCGTC |
| PkSTS  (qPCR) | ACGGAGCAATCGGTGGGAA | CCGGGATGAGCAATCCAAAACA |
| PkPMT  (qPCR) | GCCCTTTCCCGCATTCTTTCTT | TGCTGAGATTGGTAAGCCCGTAT |
| PkACC  (qPCR) | GTGGACACTGATGCACCGTA | GTCAAGCCCTGCGATGAGAT |
| PsSTS  (Cloning) | ATGTCTGTAGGAATGGGCGT | TTAAGGGAAAGGAATGCTCTTGAG |
| PsPMT  (Cloning) | ATGGAATCTGTGAAGGACGAAGTG | TTAGCACTTGATTGCTTCAATAAC |
| PsbHLH1  (Cloning) | ATGATGGATTTCATTTTCTACTTG | TTATGATGGCGGCGGCAGAG |
| PsbHLH1  (Genomic PCR) | TTCCACAGCCACCTCCACATCC | TTACTCACCGCGCTCCTCAACC |
| HPT  (Genomic PCR) | GCGTGACCTATTGCATCTCC | TTCTACACAGCCATCGGTCC |
| HPT  (RT- PCR) | GCGTGACCTATTGCATCTCC | TTCTACACAGCCATCGGTCC |
| PsbHLH1  (RT-PCR) | ATGATGGATTTCATTTTCTACTTG | TTATGATGGCGGCGG |
| PsbHLH1  (qPCR) | TGATGGGCTTCATCCCGAAG | TCTGAAATCCAACAGACCGCA |
| PsSTS  (RT-PCR) | ATGTCTGTAGGAATGGGCGT | TTAAGGGAAAGGAATGCTCTTGAG |
| PsPMT  (RT-PCR) | CTCCCCTCTGAAGATCCCGA | TGCTGAGATTGGTAAGCCCG |
| BAR  (RT-PCR) | ATGAGCCCAGAACGACGCCC | TCAGATCTCGGTGACGGGCAGGA |
| Actin  (RT-PCR) | CTTGCTGGGCGAGATTTGAC | AGCTGTCTCAAGCTCCTGTTC |
| Actin  (qPCR) | CTTGCTGGGCGAGATTTGAC | AGCTGTCTCAAGCTCCTGTTC |

**Table S1** List of primers for PCR analysis
