# Supplementary material for: A Pinus strobus transcription factor PsbHLH1 activates the production of pinosylvin stilbenoids in transgenic Pinus koraiensis calli and tobacco leaves
Source: Front Plant Sci. 2024 Jan 18;15:1342626. doi: 10.3389/fpls.2024.1342626 (PMC10830828; doi:10.3389/fpls.2024.1342626)
Supplement: Supplementary file 2 [file Presentation_1.pptx]

## Slide 1
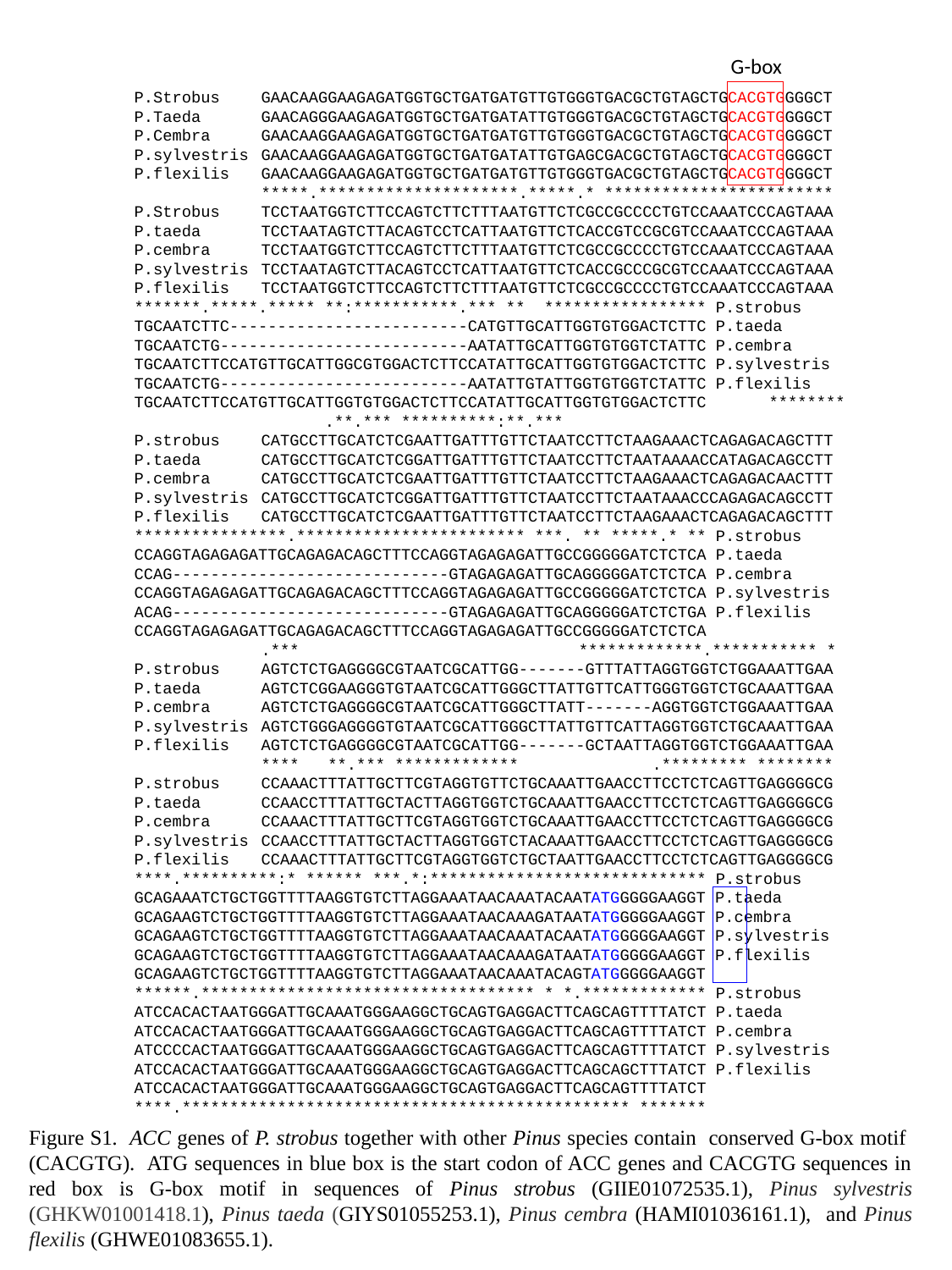

G-box
P.Strobus	GAACAAGGAAGAGATGGTGCTGATGATGTTGTGGGTGACGCTGTAGCTGCACGTGGGGCT
P.Taeda	GAACAGGGAAGAGATGGTGCTGATGATATTGTGGGTGACGCTGTAGCTGCACGTGGGGCT
P.Cembra	GAACAAGGAAGAGATGGTGCTGATGATGTTGTGGGTGACGCTGTAGCTGCACGTGGGGCT P.sylvestris	GAACAAGGAAGAGATGGTGCTGATGATATTGTGAGCGACGCTGTAGCTGCACGTGGGGCT P.flexilis	GAACAAGGAAGAGATGGTGCTGATGATGTTGTGGGTGACGCTGTAGCTGCACGTGGGGCT 	 	*****.*********************.*****.* ************************
P.Strobus	TCCTAATGGTCTTCCAGTCTTCTTTAATGTTCTCGCCGCCCCTGTCCAAATCCCAGTAAA P.taeda 	TCCTAATAGTCTTACAGTCCTCATTAATGTTCTCACCGTCCGCGTCCAAATCCCAGTAAA P.cembra 	TCCTAATGGTCTTCCAGTCTTCTTTAATGTTCTCGCCGCCCCTGTCCAAATCCCAGTAAA P.sylvestris 	TCCTAATAGTCTTACAGTCCTCATTAATGTTCTCACCGCCCGCGTCCAAATCCCAGTAAA P.flexilis 	TCCTAATGGTCTTCCAGTCTTCTTTAATGTTCTCGCCGCCCCTGTCCAAATCCCAGTAAA 	*******.*****.***** **:***********.*** ** ***************** P.strobus 	TGCAATCTTC-------------------------CATGTTGCATTGGTGTGGACTCTTC P.taeda 	TGCAATCTG--------------------------AATATTGCATTGGTGTGGTCTATTC P.cembra 	TGCAATCTTCCATGTTGCATTGGCGTGGACTCTTCCATATTGCATTGGTGTGGACTCTTC P.sylvestris 	TGCAATCTG--------------------------AATATTGTATTGGTGTGGTCTATTC P.flexilis 	TGCAATCTTCCATGTTGCATTGGTGTGGACTCTTCCATATTGCATTGGTGTGGACTCTTC 	******** .**.*** **********:**.***
P.strobus 	CATGCCTTGCATCTCGAATTGATTTGTTCTAATCCTTCTAAGAAACTCAGAGACAGCTTT P.taeda 	CATGCCTTGCATCTCGGATTGATTTGTTCTAATCCTTCTAATAAAACCATAGACAGCCTT P.cembra 	CATGCCTTGCATCTCGAATTGATTTGTTCTAATCCTTCTAAGAAACTCAGAGACAACTTT P.sylvestris 	CATGCCTTGCATCTCGGATTGATTTGTTCTAATCCTTCTAATAAACCCAGAGACAGCCTT P.flexilis 	CATGCCTTGCATCTCGAATTGATTTGTTCTAATCCTTCTAAGAAACTCAGAGACAGCTTT 	****************.************************ ***. ** *****.* ** P.strobus 	CCAGGTAGAGAGATTGCAGAGACAGCTTTCCAGGTAGAGAGATTGCCGGGGGATCTCTCA P.taeda 	CCAG-----------------------------GTAGAGAGATTGCAGGGGGATCTCTCA P.cembra 	CCAGGTAGAGAGATTGCAGAGACAGCTTTCCAGGTAGAGAGATTGCCGGGGGATCTCTCA P.sylvestris 	ACAG-----------------------------GTAGAGAGATTGCAGGGGGATCTCTGA P.flexilis 	CCAGGTAGAGAGATTGCAGAGACAGCTTTCCAGGTAGAGAGATTGCCGGGGGATCTCTCA
	.*** 	 *************.*********** *
P.strobus 	AGTCTCTGAGGGGCGTAATCGCATTGG-------GTTTATTAGGTGGTCTGGAAATTGAA P.taeda 	AGTCTCGGAAGGGTGTAATCGCATTGGGCTTATTGTTCATTGGGTGGTCTGCAAATTGAA P.cembra 	AGTCTCTGAGGGGCGTAATCGCATTGGGCTTATT-------AGGTGGTCTGGAAATTGAA P.sylvestris 	AGTCTGGGAGGGGTGTAATCGCATTGGGCTTATTGTTCATTAGGTGGTCTGCAAATTGAA P.flexilis 	AGTCTCTGAGGGGCGTAATCGCATTGG-------GCTAATTAGGTGGTCTGGAAATTGAA
	**** **.*** ************* .********* ********
P.strobus 	CCAAACTTTATTGCTTCGTAGGTGTTCTGCAAATTGAACCTTCCTCTCAGTTGAGGGGCG P.taeda 	CCAACCTTTATTGCTACTTAGGTGGTCTGCAAATTGAACCTTCCTCTCAGTTGAGGGGCG P.cembra 	CCAAACTTTATTGCTTCGTAGGTGGTCTGCAAATTGAACCTTCCTCTCAGTTGAGGGGCG P.sylvestris 	CCAACCTTTATTGCTACTTAGGTGGTCTACAAATTGAACCTTCCTCTCAGTTGAGGGGCG P.flexilis 	CCAAACTTTATTGCTTCGTAGGTGGTCTGCTAATTGAACCTTCCTCTCAGTTGAGGGGCG 	****.**********:* ****** ***.*:***************************** P.strobus 	GCAGAAATCTGCTGGTTTTAAGGTGTCTTAGGAAATAACAAATACAATATGGGGGAAGGT P.taeda 	GCAGAAGTCTGCTGGTTTTAAGGTGTCTTAGGAAATAACAAAGATAATATGGGGGAAGGT P.cembra 	GCAGAAGTCTGCTGGTTTTAAGGTGTCTTAGGAAATAACAAATACAATATGGGGGAAGGT P.sylvestris 	GCAGAAGTCTGCTGGTTTTAAGGTGTCTTAGGAAATAACAAAGATAATATGGGGGAAGGT P.flexilis 	GCAGAAGTCTGCTGGTTTTAAGGTGTCTTAGGAAATAACAAATACAGTATGGGGGAAGGT 	******.*********************************** * *.************* P.strobus 	ATCCACACTAATGGGATTGCAAATGGGAAGGCTGCAGTGAGGACTTCAGCAGTTTTATCT P.taeda 	ATCCACACTAATGGGATTGCAAATGGGAAGGCTGCAGTGAGGACTTCAGCAGTTTTATCT P.cembra 	ATCCCCACTAATGGGATTGCAAATGGGAAGGCTGCAGTGAGGACTTCAGCAGTTTTATCT P.sylvestris 	ATCCACACTAATGGGATTGCAAATGGGAAGGCTGCAGTGAGGACTTCAGCAGCTTTATCT P.flexilis 	ATCCACACTAATGGGATTGCAAATGGGAAGGCTGCAGTGAGGACTTCAGCAGTTTTATCT 	****.*********************************************** *******
Figure S1. ACC genes of P. strobus together with other Pinus species contain conserved G-box motif (CACGTG). ATG sequences in blue box is the start codon of ACC genes and CACGTG sequences in red box is G-box motif in sequences of Pinus strobus (GIIE01072535.1), Pinus sylvestris (GHKW01001418.1), Pinus taeda (GIYS01055253.1), Pinus cembra (HAMI01036161.1), and Pinus flexilis (GHWE01083655.1).
